# Spleen Stiffness Correlates with the Presence of Ascites but Not Esophageal Varices in Chronic Hepatitis C Patients

**DOI:** 10.1155/2013/857862

**Published:** 2013-08-01

**Authors:** Kazuyo Mori, Hirotaka Arai, Takehiko Abe, Hisashi Takayama, Mitsuo Toyoda, Takashi Ueno, Ken Sato

**Affiliations:** ^1^Department of Gastroenterology, Maebashi Red Cross Hospital, 3-21-36 Asahi-cho, Maebashi, Gunma 371-0014, Japan; ^2^Department of Medicine and Molecular Science, Gunma University Graduate School of Medicine, 3-39-22 Showa-machi, Maebashi, Gunma 371-8511, Japan

## Abstract

Although spleen stiffness has recently been identified as potential surrogate marker for portal hypertension, the relationship between spleen stiffness and portal hypertension has not been fully elucidated. We attempted to determine the relationship between the liver or spleen stiffness and the presence of ascites or esophageal varices by acoustic radiation force impulse (ARFI) imaging. A total of 33 chronic hepatitis C (CHC) patients (median age 68; range 51–84) were enrolled. We evaluated the relationship between the liver or spleen stiffness and indicators of portal hypertension as well as clinical and biochemical parameters. Fourteen healthy volunteers were used for validating the accuracy of AFRI imaging. The liver and spleen stiffness increased significantly with progression of liver disease. A significant positive correlation was observed between the liver and spleen stiffness. However, spleen stiffness, but not liver stiffness, was significantly associated with the presence of ascites (*P* < 0.05), while there was no significant association between the spleen stiffness and spleen index/presence of esophageal varices in CHC patients. The area under the receiver operating characteristic curve based on the spleen stiffness was 0.80. In conclusion, spleen stiffness significantly correlates with the presence of ascites but not esophageal varices in CHC patients.

## 1. Introduction

In cirrhotic patients with portal hypertension, increase of the spleen size is due mainly to tissue hyperplasia associated with a parallel increase of the splenic blood flow, which probably participates in the pathogenesis of portal hypertension [1]. However, the role of spleen stiffness among the spleen changes associated with portal hypertension has not been fully elucidated. Recently, liver stiffness has been measured easily using noninvasive modalities including transient elastography (TE), MR elastography, and acoustic radiation force impulse (ARFI) imaging. Although a significant correlation between liver stiffness as assessed by TE and portal hypertension as evaluated by hepatic venous pressure gradient (HVPG) measurement has been reported [2–6], only several studies have applied these techniques to measurement of the spleen stiffness. To the best of our knowledge, there are reports [6–10] of evaluation of the relationship between the spleen stiffness and the presence of portal hypertension by different noninvasive modalities, that is, TE, MR elastography, and ARFI imaging. These studies evaluated the relationship between the spleen stiffness and the presence of esophageal varices as an indicator of portal hypertension. However, the results were contradictory. On the other hand, there has been no evaluation of the relationship between spleen stiffness and the presence of ascites as another indicator of portal hypertension.

ARFI imaging is a new elastographic technology integrated into conventional B-mode ultrasonography. It is an inexpensive and noninvasive modality, conveniently performed in patients who are not suitable candidates for MRI. ARFI imaging allows quantification of the shear-wave velocity (SWV) (m/sec), which has been shown to be correlated with the liver fibrosis stage [11–13]. The higher the tissue stiffness, the greater the SWV (m/sec). The longitudinal waves from the push pulses are transmitted through ascites, whereas the shear waves are measured only in the region of interest. Therefore, SWV assessment is possible in the presence of ascites, differing from the measurement by TE [11]. In addition, liver stiffness by ARFI imaging is comparable to that by TE from the point of view of diagnostic accuracy and is unaffected by the serum ALT levels, again differing from the measurement by TE [12]. In this study, we evaluated the relationship between liver or spleen stiffness as assessed by ARFI imaging and the presence of ascites or esophageal varices as indicators of portal hypertension.

## 2. Patients and Methods

### 2.1. Patients and Study Design

Thirty-three consecutive hepatitis C patients (median age 68; range 51–84) with chronic hepatitis or liver cirrhosis seen at our institution were enrolled. The control group, which was used for validating the accuracy of ARFI imaging, was composed of 14 healthy volunteers (median age 33; range 24–52). The median age was lower in the control group than in the chronic hepatitis group or liver cirrhosis group. The inclusion criteria for the controls were at the age of 18 years or older, no history of chronic liver disease, and normal serum liver enzyme levels at the time of enrollment.

Laboratory tests, including the serum bilirubin and albumin, platelet count, prothrombin time, and serum markers of fibrosis (type IV collagen, hyaluronate), were performed in all patients within 1 month from the beginning of the study. In all patients, a fiberoptic upper gastrointestinal endoscopy was performed to check for the presence of esophageal varices and abdominal ultrasonography to check for the presence of ascites and determine the spleen index. Esophageal varices were evaluated by the endoscopist without knowledge of the data of liver and spleen stiffness measurement. Calculation of the median liver and spleen stiffness was possible in all subjects. Liver cirrhosis was diagnosed by the predictive model of cirrhosis in patients with CHC based on standard laboratory tests [14]. This study was conducted with the approval of the institutional ethics committee. Informed consent was obtained from all the volunteers and all the patients, in accordance with the principles of the Declaration of Helsinki.

### 2.2. Liver and Spleen Stiffness Measurement

Liver and spleen stiffness were measured by ARFI imaging in Virtual Touch Tissue Quantification using an ACUSON S2000 apparatus (Mochida SIEMENS Medical System, Tokyo). One experienced sonographer operated the apparatus. ARFI imaging involves targeting an anatomic region to be interrogated for elastic properties with the use of a region-of-interest (ROI) cursor while performing real-time B-mode imaging. Transmission of longitudinal acoustic pulses leads to tissue displacement, which results in shear-wave propagation away from the region of excitation. The SWV is measured within a defined ROI (central window of 5 mm axial by 4 mm width) by using ultrasound tracking beams laterally adjacent to the single push beam. The shear-wave propagation velocity is proportional to the square root of the tissue elasticity. Results are expressed in meters per second. We used a curved array at 4 MHz for B-mode ARFI imaging. We measured the liver stiffness in the right liver robe, 2-3 cm below the liver capsule, via an intercostal approach, and the spleen stiffness in the lower pole of the spleen, 2-3 cm below the spleen capsule, also via an intercostal approach. In each patient, 5 valid ARFI imaging measurements were performed in the liver and the spleen based on previous reports [12, 13, 15]. Furthermore, the median values were calculated, the results being expressed as SWV (m/sec). Measurement failure was defined as zero valid shots like TE, and unreliable measurements were defined as an interquartile range (IQR) to median value ratio greater than 30% or a success rate less than 60% [12, 16].

### 2.3. Statistical Analysis

All data were presented as median and range. Analysis of variance for comparison of more than two groups was performed using the Kruskal Wallis *H*-test followed by Mann-Whitney's *U*-test with post-hoc Bonferroni's correction. Analysis of variance for comparison of two groups was performed by Mann-Whitney's *U*-test. Comparisons of percentages between the ascites and non-ascites groups or between the esophageal varices and non-varices groups were performed using Fisher's exact test. The correlations between the liver stiffness and the spleen stiffness or between the spleen index and spleen stiffness were assessed by calculation of Spearman's correlation coefficient. We assessed the diagnostic performance of ARFI imaging by receiver operating characteristic curve (ROC curve) analysis. The ROC curve represents the sensitivity versus (1-specificity) for all possible cut-off values. Area under the ROC curve (Az) and 95% confidence intervals of the Az values were calculated. Differences were regarded as significant when the *P* values were less than 0.05.

## 3. Results

### 3.1. Characteristics of Study Subjects

The clinical and biochemical characteristics of the study subjects are listed in Table 1. Among the 24 cirrhotic patients, 8 (33.3%) were classified as Child-Pugh class A, 10 (41.7%) as Child-Pugh class B, and 6 (25.0%) as Child-Pugh class C. Esophageal varices and ascites were present in 12 (50.0%) and 15 (62.5%) patients, respectively.

### 3.2. Liver Stiffness in Healthy Volunteers and Patients with Chronic Hepatitis and Liver Cirrhosis

**Figure 1:**
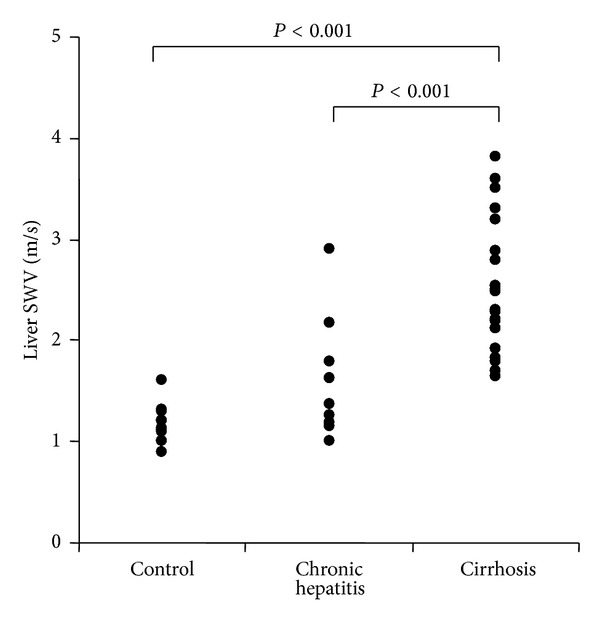
Liver shear-wave velocity (SWV; m/sec) in healthy volunteers and patients with chronic hepatitis and liver cirrhosis.

Determination of liver stiffness was possible in all volunteers and patients. There were no subjects who met the definition of unreliable measurements of liver stiffness.  Figure 1 shows the liver stiffness as determined by ARFI imaging in the control, chronic hepatitis, and liver cirrhosis groups. The median liver SWV (range) values were 1.17 m/sec (IQR 1.03–1.21 m/sec), 1.36 m/sec (IQR 1.19–1.76 m/sec), and 2.4 m/sec (IQR 2.07–2.97 m/sec), respectively. The liver stiffness differed significantly between each two of the three groups. The *P* values of the significant differences between the groups were as follows: control versus cirrhosis (*P* < 0.001); chronic hepatitis versus cirrhosis (*P* < 0.001).

### 3.3. Spleen Stiffness in Healthy Volunteers and Patients with Chronic Hepatitis and Liver Cirrhosis

**Figure 2:**
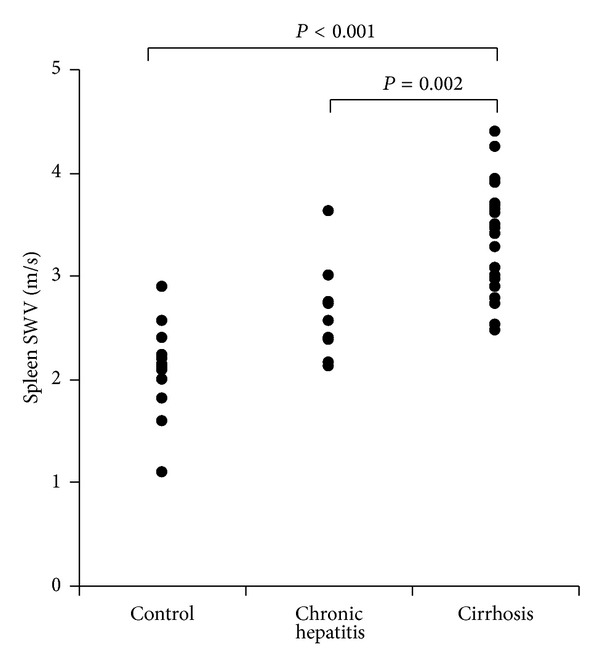
Spleen shear-wave velocity (SWV; m/sec) in healthy volunteers and patients with chronic hepatitis and liver cirrhosis.

Determination of spleen stiffness was possible in all volunteers and patients. There were no subjects who met the definition of unreliable measurements of spleen stiffness.  Figure 2 shows the spleen stiffness as determined by ARFI imaging in the control, chronic hepatitis, and liver cirrhosis groups. The median spleen SWV values (range) were 2.12 m/sec (IQR 2.00–2.23 m/sec), 2.57 m/sec (IQR 2.38–2.75 m/sec), and 3.40 m/sec (IQR 2.95–3.66 m/sec), respectively. The spleen stiffness differed significantly between each two of the three groups. The *P* values of the significant differences between the groups were as follows: control versus cirrhosis (*P* < 0.001); chronic hepatitis versus cirrhosis (*P* = 0.002). 

### 3.4. Correlation between the Liver Stiffness and Spleen Stiffness in Healthy Volunteers and Patients with Chronic Hepatitis and Liver Cirrhosis

**Figure 3:**
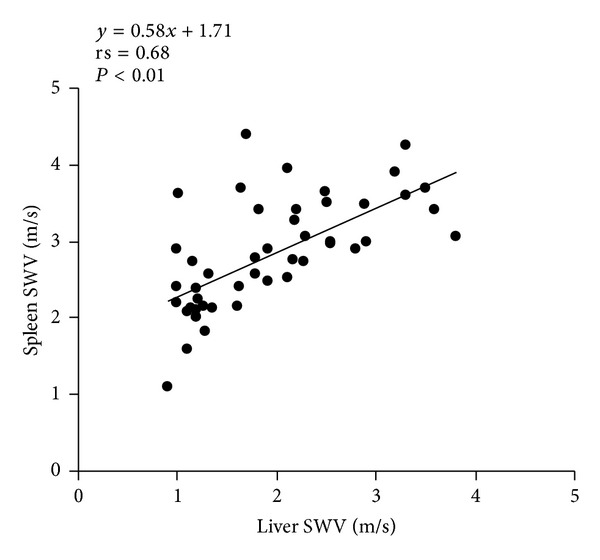
Correlation between the liver shear-wave velocity (SWV; m/sec) and the spleen shear-wave velocity (SWV; m/sec) in healthy volunteers and patients with chronic hepatitis and liver cirrhosis. rs: Spearman's rank correlation coefficient.

Figure 3 shows the correlation between the liver stiffness and spleen stiffness. In the overall subject population, there was a statistically significant positive correlation between the liver stiffness and spleen stiffness (rs = 0.68, *P* < 0.01).

### 3.5. Correlation between the Spleen Stiffness and Spleen Index in Healthy Volunteers and Patients with Chronic Hepatitis and Liver Cirrhosis

In the overall subject population, no significant correlation was found between the spleen stiffness and spleen index.

### 3.6. Relationship between the Presence of Ascites and the Liver/Spleen Stiffness in Patients with Liver Cirrhosis

**Figure 4:**
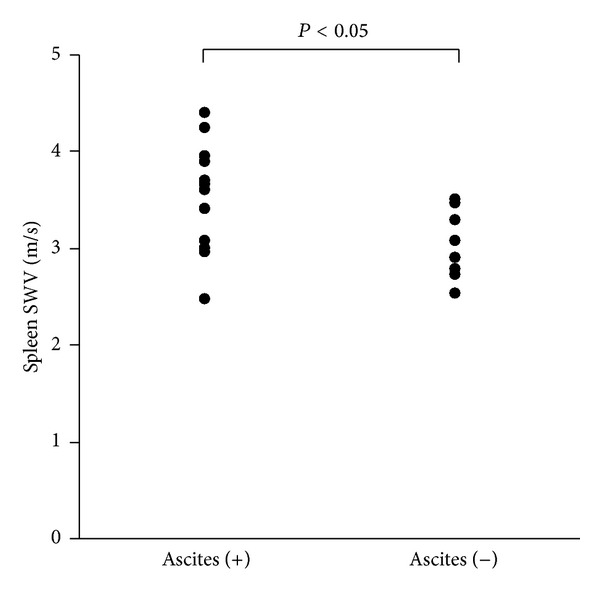
Spleen shear-wave velocity (SWV; m/sec) in patients with liver cirrhosis.

The median liver SWV (range) was 2.54 m/sec (IQR 2.02–3.30 m/sec) in the presence of ascites and 2.28 m/sec (IQR 2.12–2.50 m/sec) in the absence of ascites. The liver stiffness did not differ between the groups with and without ascites. On the other hand, the median spleen SWV (range) was 3.6 m/sec (IQR 3.24–3.80 m/sec) in the presence of ascites and 2.90 m/sec (IQR 2.78–3.28 m/sec) in the absence of ascites (Figure 4). The spleen stiffness was significantly elevated in the group with ascites as compared with that in the group without ascites (Figure 4) (*P* < 0.05).

### 3.7. Relationship between the Presence of Esophageal Varices and the Liver/Spleen Stiffness in Patients with Liver Cirrhosis

The median liver SWV (range) was 2.39 m/sec (IQR 1.89–2.63 m/sec) in the presence of esophageal varices and 2.40 m/sec (IQR 2.12–2.90 m/sec) in the absence of esophageal varices. Also, the median spleen SWV (range) was 3.41 m/sec (IQR 3.04–3.66 m/sec) in the presence of esophageal varices and 3.18 m/sec (IQR 2.90–3.63 m/sec) in the absence of esophageal varices. The liver stiffness and spleen stiffness did not differ between the groups with and without esophageal varices.

### 3.8. Clinicopathological Factors Associated with the Presence of Ascites in Patients with Liver Cirrhosis

The clinical characteristics of the patients with cirrhosis are shown in Table 2. In the univariate analysis, the prothrombin time (*P* = 0.012) and spleen stiffness (*P* = 0.012) were found to be significantly different between the cirrhotic patients with and without ascites. 

### 3.9. Determination of Liver/Spleen Stiffness Cut-Off Values for Detecting Ascites in Patients with Liver Cirrhosis

The liver stiffness cut-off value of 2.52 m/sec had a negative predictive value (NPV) of 50.0%, positive predictive value (PPV) of 80.0%, sensitivity of 53.3%, specificity of 77.8%, accuracy of 62.5%, positive likelihood ratio (+LR) of 2.40, and negative likelihood ratio (–LR) of 1.67. In contrast, the spleen stiffness cut-off value of 3.34 m/sec had an NPV of 63.6%, PPV of 84.6%, sensitivity of 73.3%, specificity of 77.8%, accuracy of 75.0%, +LR of 3.30, and −LR of 2.92. 

**Figure 5:**
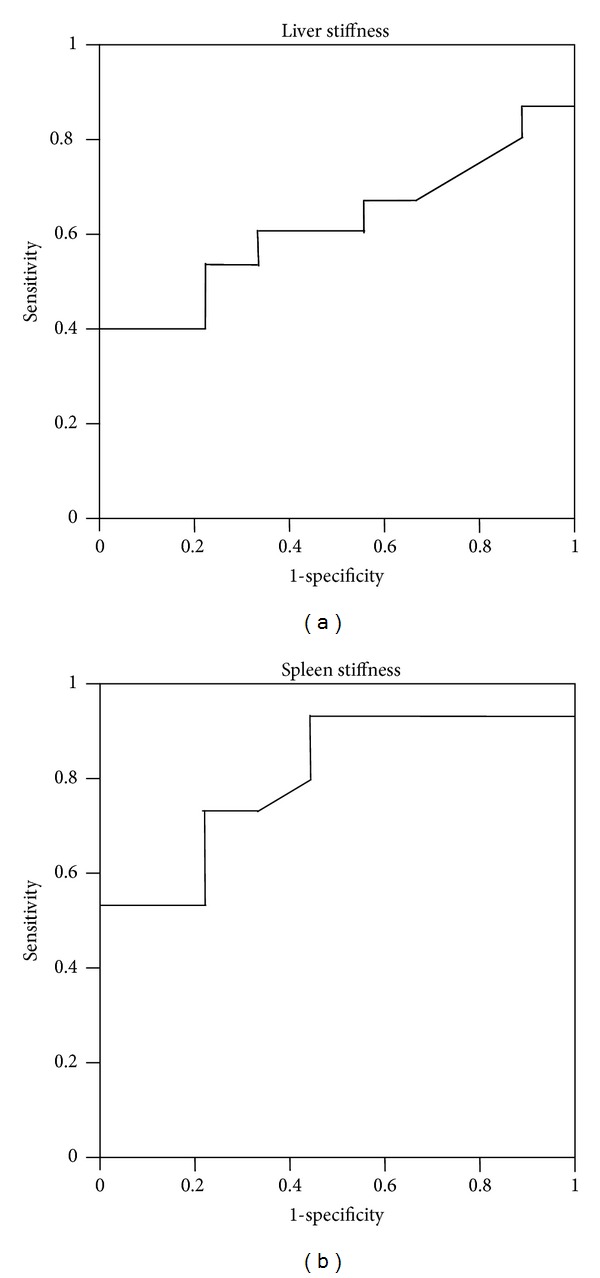
Receiver operating characteristic (ROC) curve showing the correlation between the presence of ascites and liver shear-wave velocity (SWV; m/sec) (a) or spleen shear-wave velocity (SWV; m/sec) (b) as assessed by acoustic radiation force impulse (ARFI) imaging in patients with liver cirrhosis. The ideal area under the curve is 1.00. The solid lines represent ROC curves based on the liver stiffness (a) and spleen stiffness (b).

Figure 5 shows ROC that is the prediction with complication of ascites with ARFI imaging in the patients with liver cirrhosis. The area under the ROC curves based on liver stiffness (a) and spleen stiffness (b) were 0.62 (0.11 standard error; 95% confidence index 0.39–0.84) and 0.80 (0.09 standard error; 95% confidence index 0.63–0.98), respectively. 

## 4. Discussion

The major findings of our study were that the spleen stiffness, but not the liver stiffness, was correlated with the presence of ascites and that the spleen stiffness did not correlate with the presence of esophageal varices in chronic hepatitis C patients. We also showed that ARFI imaging is an excellent modality for measurement of not only the liver stiffness but also the spleen stiffness, even in the presence of ascites. Our study is the first report until now to evaluate the association between spleen stiffness and the presence of ascites. Ascites can be relatively easily detected by conventional abdominal ultrasonography. However, the finding is of potential clinical significance in view of the possibility of predicting the development of ascites in chronic liver disease by measuring the spleen stiffness, even before ascites can be detected by conventional abdominal ultrasonography. Specifically, ARFI imaging might be useful to predict the development of ascites in posttransarterial chemoembolization by measuring the spleen stiffness before transarterial chemoembolization. In addition, in the event of difficulty in detecting the presence of ascites by conventional ultrasonography due to miscellaneous reasons, such as noticeable obesity or posthepatic resection, ARFI imaging may be useful for suggesting the presence of ascites by measuring the spleen stiffness in an inexpensive and noninvasive way. Previous studies reported that the rates of unsuccessful measurements for liver and spleen stiffness using ARFI imaging were 2.8% [17] and 4.8% [8], respectively. In our study, we could detect liver and spleen stiffness in all volunteers and patients. Because the number of our study subjects is relatively small, we believe that our results are consistent with these data [8, 17].

One of the possible reasons for the finding that the liver stiffness was not significantly correlated with the presence of ascites is as follows. Portal hypertension develops as a result of an increase in intrahepatic resistance to portal blood flow due to the profound morphologic changes in the liver characterized by fibrosis and regenerative nodules compressing the sinusoids, which lead to vascular obliteration, activation of the hepatic stellate cells, and vasoconstriction due to intrahepatic nitric oxide deficiency and enhanced vasoconstrictor activity [3]. In the advanced stage of cirrhosis, several extrahepatic factors such as the hyperdynamic circulation, splanchnic vasodilatation, and resistance to portal blood flow posed by the portosystemic collaterals contribute to the rise in the portal pressure [18]. Thus, the liver stiffness may not be sufficient for reflecting the complex hemodynamic changes characteristic of the impending portal hypertension. 

One of the possible reasons for the positive correlation between the spleen stiffness and presence of ascites is as follows. The anatomic features and microcirculation of the spleen are well characterized. Splenic tissue is composed primarily of red pulp tissue and, to a lesser degree, white pulp. Within the red pulp, blood is received by the penicillar arterioles, which open directly into the venous sinuses and trabecular vein. Blood exits through the splenic vein into the splanchnic venous circulation. White pulp is composed of a central artery surrounded by lymphoid tissue. Penicillar arterioles originate from the central arteries outside the white pulp and drain into the venous sinuses and the red pulp [7]. Thus, portal hypertension leads to pulp hyperplasia, which may increase the spleen stiffness. If this hypothesis were true, the spleen stiffness would be correlated with the spleen index. However, there was no correlation between the spleen stiffness and spleen index in our study. To the best of our knowledge, a consistent relation between splenomegaly and portal venous pressure has not yet been identified [19–21]. Splenomegaly is related not only to blood congestion as a consequence of increased portal pressure and augmented resistance to splenic vein outflow but also to multiple other histopathologic changes, such as arterial aneurysms [22] and concern with endothelin [23], and the immunologic pathway from the liver [24]. Therefore, it may not be valid to simply replace the spleen index by the spleen stiffness. 

No significant correlation was found between the presence of esophageal varices and the liver stiffness or spleen stiffness in our study. This was probably because esophageal varices are only one of the components of collateral circulation and represent only a part of portal hypertension. Thus, the correlation may depend on the collateral circulation dynamics in the study population. In fact, previous reports on the association between esophageal varices and the liver stiffness or spleen stiffness are inconsistent. Some studies showed that the liver stiffness was positively correlated with the presence of esophageal varices [6, 10] and that spleen stiffness predicts or is associated with the presence [6, 7, 9] or grade of esophageal varices [6]. However, no correlation was seen between the grade or size of the esophageal varices and the liver stiffness or spleen stiffness [2, 9]. Bota et al. reported that the spleen stiffness as assessed by ARFI imaging could not predict the presence or severity of esophageal varices [8], lending support to our data. These contradictory findings may support our hypothesis; that is, the correlation may depend on the collateral circulation dynamics in the study population.

Previous studies with ARFI imaging [7, 8] showed a systematic association between the stage of fibrosis and the liver stiffness. Bota et al. [8] showed that the spleen stiffness increased with progression of the stage of liver disease. Talwalkar et al. [7] showed that the liver stiffness was significantly correlated with the spleen stiffness. Our data were consistent with these reports.

Our study had limitations. First, we did not measure HVPG as the portal venous pressure; therefore, the correlation between the spleen stiffness and the portal venous pressure could not be evaluated. Another limitation was the relatively small sample size. 

## 5. Conclusions

Spleen stiffness, but not liver stiffness, was positively correlated with the presence of ascites as a representative marker of portal hypertension in patients with liver cirrhosis and the spleen stiffness did not correlate with the presence of esophageal varices in chronic hepatitis C patients. AFRI imaging showed the advantage of feasibility regardless of the presence of ascites, differing from the measurement by TE, and was applicable in patients who were unsuitable candidates for MRI, differing from the measurement by MRI elastography. However, our study is only preliminary due to the relatively small sample size, and a larger cohort study to verify our results is warranted in the future.

## Figures and Tables

**Table 1:** Clinical and laboratory findings.

	Healthy volunteers (*n* = 14)		Patients	
		All (*n* = 33)	Chronic hepatitis (*n* = 9)	Cirrhosis (*n* = 24)
Age (y)*	32 (25–41)	67 (51–84)	65 (51–73)	67.5 (53–84)
Male/female	5/9	17/16	4/5	13/11
Bilirubin (mg/dL)*		1.0 (0.4–3.9)	0.5 (0.6–1.2)	1.3 (0.4–3.9)
Albumin (g/dL)*		3.4 (2.4–4.5)	4.3 (3.5–4.5)	3.0 (2.4–3.9)
Platelet count (10^7^/L)*		10.3 (3.5–21.5)	15.4 (10.3–21.5)	8.4 (3.5–14.3)
Prothrombin time (%)*		69 (39–100)	77 (64–100)	66.5 (39–84)
Type IV collagen (ng/mL)*		217 (100–681)	134 (100–255)	229 (126–681)
Hyaluronate (ng/mL)*		454 (29–2860)	68 (29–627)	512 (245–2860)
Ascites				
+		15	0	15
−		18	9	9
Varices				
+		12	0	12
−		21	9	12
Child-Pugh score				
Grade A				8
Grade B				10
Grade C				6

Note. *Results are expressed as median (range).

**Table 2:** Clinical characteristics of the patients with cirrhosis.

	Patients with ascites (*n* = 15)	Patients without ascites (*n* = 9)	*P* value
Bilirubin (mg/dL)	1.4 (0.4–3.9)	1.1 (0.4–1.7)	0.064
Albumin (g/dL)	2.8 (2.5–4.1)	3.5 (2.4–4.0)	0.055
Platelet count (10^7^/L)	8.3 (3.5–12.7)	10.4 (3.9–14.3)	0.12
Prothrombin time (%)	60 (39–100)	70 (59–90)	0.012
Type IV collagen (ng/mL)	238 (126–681)	223 (144–247)	0.15
Hyaluronate (ng/mL)	724 (82–2860)	440 (263–728)	0.047
Shear wave velocity, liver (m/sec)	2.54 (1.65–3.81)	2.28 (1.79–2.89)	0.38
Shear wave velocity, spleen (m/sec)	3.41 (2.47–4.41)	2.90 (2.52–3.50)	0.012
Spleen index	40.5 (11.3–122.5)	51.8 (45.8–79.8)	0.23

Note. Results are expressed as median (range).
